# Exercise modulates polarization of TAMs and expression of related immune checkpoints in mice with lung cancer

**DOI:** 10.7150/jca.76136

**Published:** 2022-09-06

**Authors:** Zhe Ge, Shan Wu, Zhengtang Qi, Shuzhe Ding

**Affiliations:** 1 School of Sport, Shenzhen University, Shenzhen 518060, China.; 2 Key Laboratory of Adolescent Health Assessment and Exercise Intervention of Ministry of Education, East China Normal University, Shanghai 200241, China.

**Keywords:** exercise, TAMs, PD-L1, CD47, CD24, Lung cancer, IL-10/12

## Abstract

**Purpose:** Many studies have found that both endurance exercise (EX) and high-intensity interval training (HIIT) have a positive therapeutic effect on the treatment of lung cancer patients, but the specific mechanism is unclear. Therefore, we investigated whether EX and HIIT could delay the progression of lung cancer by affecting the infiltration of tumor-associated macrophages (TAMs) and restoring the tumor phagocytic activity of TAMs in lung cancer tissue.

**Methods:** BALB/c mice were divided into 4 groups. The mice were given saline as the saline group (Saline), and the mice were given urethane as the lung cancer mice. The lung cancer mice were randomly divided into the control group (CON), EX group, and HIIT group. After exercise, the cancer tissues were collected for RT-PCR, immunofluorescence staining, and Wes automated western blotting system analysis.

**Results:** Compared with the Saline group, the mRNA levels of TAMs M1 markers IL-6, TNF-α, iNOS, and M2 markers CD206, IL-10, and Arg-1 in the CON group were significantly increased (P<0.05). There was no significant difference in the percentage of F4/80 positive cells among the groups. Compared with the CON group, the percentage of CD86-positive cells in TAMs in the EX group was significantly decreased (P<0.05). From the protein expression level, compared with the CON group, the expression of SIRPα in the EX group was significantly increased (P<0.0001) and the expression of PD-L1 had a tendency to increase (P=0.06). Compared with the CON group, the expressions of IL-10, IL-12, CD47, and CD24 in the HIIT group were significantly increased (P<0.05). In addition, compared with the CON group, plasma IFN-γ in the EX group and HIIT group was significantly increased (P<0.05).

**Conclusion:** Lung cancer tissue presents an inflammatory tumor microenvironment. The therapeutic effect of exercise on lung cancer is independent of the infiltration of TAMs in lung cancer tissue. In addition, endurance exercise can reduce the proportion of M1-type TAMs in lung cancer tissues, while HIIT antagonistically regulates M1 and M2 polarization of TAMs by increasing the levels of IL-10 and IL-12 in lung cancer tissues and circulating IFN-γ. Finally, endurance exercise and HIIT can modulate the expression of some immune checkpoints in lung cancer tissues.

## Introduction

Cancer is one of the principal diseases that threaten human life and health in the world. Nowadays, the global cancer incidence and death rate are rapidly growing worldwide 1. Lung cancer is among the most common malignant tumors in the world. According to the global cancer statistics in 2020, the mortality rate of lung cancer ranks first among all major cancers 2. Macrophages play their role in immune surveillance to destroy tumor cells through phagocytosis. However, tumor cells not only inhibit the tumor-killing activity of macrophages but also utilize macrophages to support their growth. For example, tumor tissues are infiltrated with a large number of macrophages, the so-called tumor-associated macrophages (TAMs), in which most of these macrophages exhibit the morphology of M2 macrophages. Moreover, tumor cells also directly hamper the TAMs phagocytosis through immune checkpoints, such as CD47/signal regulatory protein-α (SIRPα), CD24/sialic acid binding Ig-like lectin 10 (SIGLEC10), major histocompatibility complex class I molecules (MHC-I)/leukocyte immunoglobulin-like receptor subfamily B member 1 (LILRB1), and PD-1 ligand 1 (PD-L1)/programmed cell death protein-1 (PD-1) axis 3. In addition, TAMs also support tumor growth and metastasis through various strategies. For example, TAMs promote cancer cell proliferation and cancer stemness by secreting cytokines 4, 5. In addition, it can also strengthen the immune escape of tumor cells 6. Notably, regular aerobic exercise can improve the anti-tumor activity of macrophages 7. Moreover, prolonged aerobic exercise suppresses the lung metastasis of melanoma and enhances the tumor cytotoxicity of alveolar macrophages 8. In addition, high-intensity interval exercise also enhances the ability of macrophages to engulf and kill tumor cells 9. Of note, M2-to-M1 repolarization of TAMs can restore the anti-tumor effects of TAMs 10, 11. These studies show that exercise can potentiate the anti-tumor activity of macrophages, in which this process may be related to the polarization conversion of TAMs and the interaction between tumor cells and TAMs. Many studies have found that both endurance exercise and high-intensity interval exercise have a positive therapeutic effect on the treatment of lung cancer (Table 1), but whether this is related to TAMs remains unclear. Therefore, we further explored the infiltration and polarization of TAMs and their receptor-ligand interactions with tumor cells in lung cancer tissues under the regulation of endurance exercise and HIIT, respectively, to provide a new therapeutic strategy for the treatment of lung cancer patients.

## Material and Methods

### Animals and groups

6-week-old specific-pathogen-free (SPF) grade BALB/c female mice were purchased from the Experimental Animal Center of East China Normal University. The lung cancer mice were derived from a previously established mouse model of lung cancer 15. All mice were housed in a temperature-controlled (21-22℃) SPF laboratory animal room under 12-hr light-dark cycles with access to food and water ad libitum. BALB/c female mice were the healthy control group (Saline group, n=10). Lung cancer mice were randomly given rest (CON group, n=15), endurance exercise (EX, n=15), and high-intensity interval training (HIIT, n=15).

### Exercise Model

Lung cancer model mice in the EX group and the HIIT group performed 12 weeks of exercise. The EX group performed endurance exercise with an exercise intensity of 15m/min (80% VO2max) for 45 minutes, 5 times a week 18. The HIIT group performed high-intensity interval swimming exercises with a plastic water tank of 40 x 30 x 80 cm and a water temperature of 30±2°C. A lead mass of 10% or 12% of body weight was suspended from the tail of the mouse, forced to swim for 20 seconds, and then passively recovered for 10 seconds, repeated 10 times, 4 times a week. The percentage of load weight was gradually increased, 10% in the first 6 weeks and 12% in the last 6 weeks 19.

### RNA extraction and real-time quantitative PCR

Total RNA was extracted from frozen tissue with the use of TRIZOL (Invitrogen) according to the manufacturer's specifications. The total RNA extracted was used 1ug for reverse transcription synthesis of cDNA. ReverTra Ace qPCR RT Kit (TOYOBO, Osaka, Japan) was used for reverse transcription. Fluorescence quantitative PCR was performed using ABI QuantStudio 3 real-time fluorescence quantitative PCR instrument and software (ABI, California, USA) and Hieff® qPCR SYBR Green Master Mix (Low Rox Plus) (yeasen, China). All primers were synthesized by Sangon (Shanghai, China). The remaining target genes were standardized by 18S rRNA. All the data were analyzed by 2 -ΔΔCT method. Primers used in the study are as follows:

CD86, 5'-GCAGCACGGACTTGAACAAC-3'and 5'-CCTTTGTAAATGGGCACGGC-3'; CD206, 5'-GCACTGGGTTGCATTGGTTT-3' and 5'-CCTGAGTGGCTTACGTGGTT-3'; iNOS, 5'-AAGCGCAAAACATTTCCTGGG-3'and 5'-CACATACTGTGGACGGGTCG-3'; IL-6, 5'-CCCCAATTTCCAATGCTCTCC-3'and 5'-CGCACTAGGTTTGCCGAGTA-3'; TNF-α, 5'-CCCTCACACTCACAAACCAC-3' and 5'-ATAGCAAATCGGCTGACGGT-3'; IL-12p40, 5'-CGCCACACAAATGGATGCAA-3' and 5'-TGTGTCCTGAGGTAGCCGTA-3'; IL-10, 5'-GCTCCAAGACCAAGGTGTCT-3' and 5'-CGGAGAGAGGTACAAACGAGG-3'; Arg-1, 5'-CGGGAGGGTAACCATAAGCC-3' and 5'-CTTGGGAGGAGAAGGCGTTT-3'; H2-Kd, 5'-CAGTCCACCCCTCTACACCA-3' and 5'-ACAAGAAATCAGCCCTAGGTCA-3'; PD-1, 5'-AACCAGAAGGCCGGTTTCAA-3' and 5'-AGTGTCGTCCTTGCTTCCAG-3'; PD-L1, 5'-ACTTGCTACGGGCGTTTACT-3' and 5'-AGGGCAGCATTTCCCTTCAA-3'; CD47, 5'-CCTGTCCCGTTCTGCTACTT-3' and 5'-TGCTTTGTCATGCCTCCGAT-3'; SIRPα, 5'-TCACCCGAAACCATACCGTG-3' and 5'-TGTGTCCTGGATCAAAGACTGT-3'; CD24, 5'-TTCGCATGGTCACACACTGA-3' and 5'-ACACACACAGTAGCTTCGGG-3'; F4/80, 5'-TGTCTGAAGATTCTCAAAACATGGA-3' and 5'-TGGAACACCACAAGAAAGTGC-3'; 18S, 5'-AGCTTGCGTTGATTAAGTCCCT-3' and 5'-GCCTCACTAAACCATCCAATCGG-3'.

### Immunofluorescence

Mouse lung tumor tissues were made into paraffin-embedded sections. For immunofluorescence staining, slides were fixed with 4% paraformaldehyde, permeabilized with 0.15% Triton X-100, blocked with 3% BSA for 30 min at room temperature (RT), and incubated with primary antibodies (F4/80, GB11027, Servicebio, 1:4000; CD24, DF8518, affinity, 1:100) in phosphate-buffered saline. Then, slides were incubated with secondary antibodies (Cy3-Goat anti-rabbit, GB21303, Servicebio, 1:300). For immunofluorescence double staining, slides were incubated with primary antibodies (F4/80, GB11027, Servicebio, 1:4000; CD86, DF6332, affinity, 1:200; CD206, GB13438, Servicebio, 1:500) in phosphate-buffered saline. Then, slides were incubated with secondary antibodies (HRP-Goat anti-rabbit, GB23303, Servicebio, 1:500; Cy3-Goat anti-rabbit, GB21303, Servicebio, 1:300) and FITC-Tyramide (G1222-50UL, servicebio). After staining, sections were placed under a microscope (Nikon Eclipse E100, Tokyo, Japan) for observation. Images were analyzed using Image-Pro Plus 6.0 software.

### Wes automated western blotting system

Tissue protein extraction was performed using membrane, nuclear and cytoplasmic protein extraction kit (Sangon Biotech, China) according to the kit instructions. The separated cytoplasm and cell membrane proteins were used by the BCA protein assay kit to determine protein concentration (Beyotime, China). The samples were detected using the Wes^TM^ automatic protein expression analysis system (ProteinSimple, USA), and the specific steps were carried out following the instrument manual. Due to Wes-ProteinSimple, the following primary antibodies were used:

Primary rabbit polyclonal antibodies to CD86 (DF6332, affinity), CD47 (DF6649, affinity), CD24 (DF8518, affinity), CD274 (DF6526, affinity), SIRPα (AF0253, affinity), CD206 (DF4149, affinity), PD-1 (DF3699, affinity), ATP1A1 (AF6083, affinity), IL-12 (DF2519, affinity), IL-10 (DF6894, affinity) and mouse monoclonal antibodies to Qa-1 (sc-23889, santa), H2-K^d^ (sc-53852, santa), β-acting (sc47778, santa). Moreover, primary antibodies were used in combination with HRP-conjugated secondary antibodies (Jackson). The membrane reference protein is ATP1A1, and the cytoplasmic reference protein is β-acting.

### ELISA

The plasma IFN-γ (Shanghai mlbio), IL-12 (Shanghai mlbio) and IL-10 (Shanghai mlbio) levels were analyzed according to the manufacturers' instructions.

### Statistical analysis

Independent student's t-test was used for the data collected only from Saline group and CON group. Statistical analysis for multiple comparisons was performed in GraphPad Prism 7 using a one-way ANOVA followed by Dunnett's many-to-one test. The data were represented by mean ± SEM. Note: &, && respectively represent P<0.05, P<0.01 compared with Saline group. *, **, ***, **** respectively represent P<0.05, P<0.01, P<0.001, P<0.0001 compared with CON group.

## Result

### The delay of tumor progression caused by exercise is independent of the infiltration of tumor tissue TAMs

**Figure 1:**
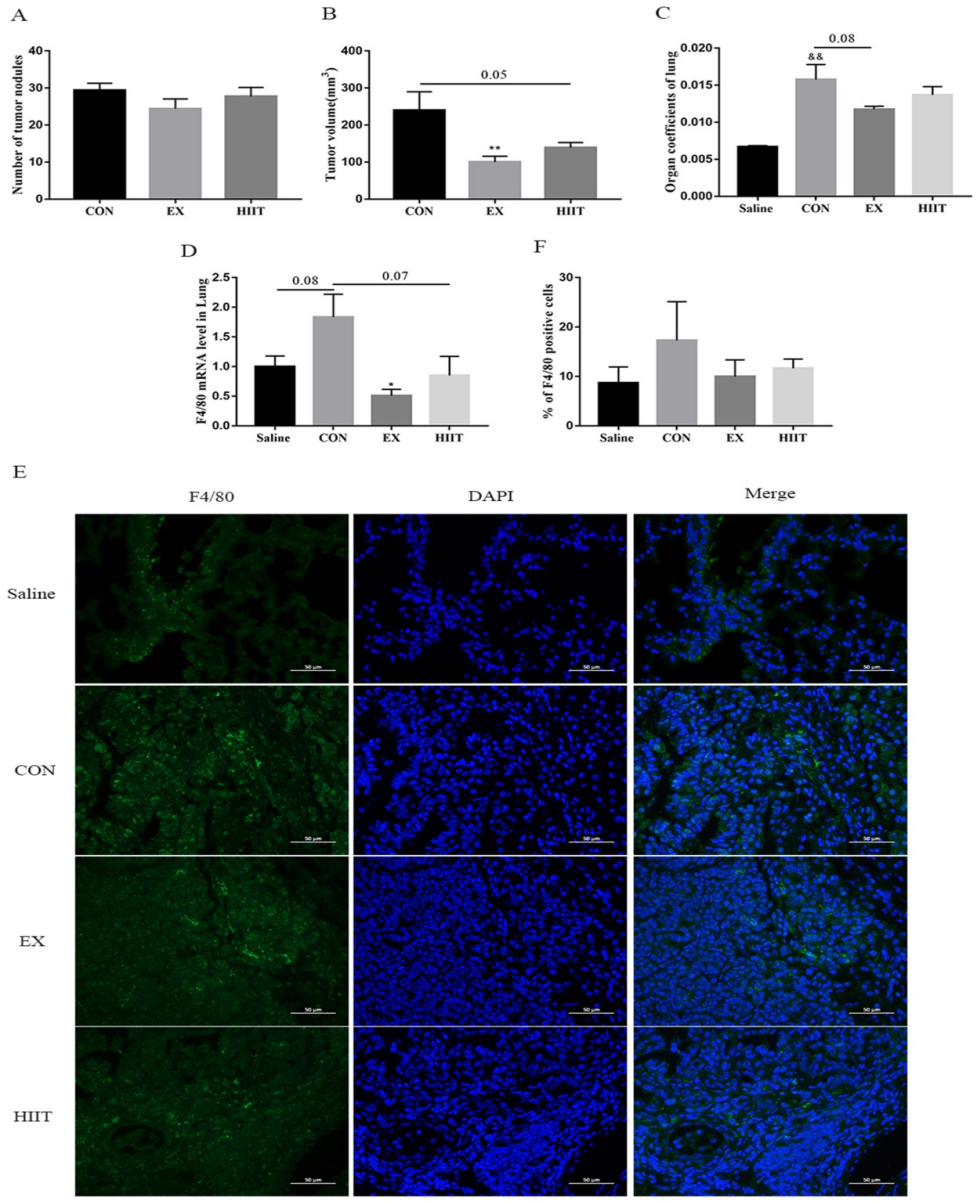
Effects of exercise on tumor growth and infiltration of TAMs in mice with lung cancer. A, B: The effect of exercise on the number of tumor nodules (A) and tumor size (B) in mice with lung cancer (n=10). C: The effect of exercise on lung tissue organ coefficient (n=8). D: The effect of exercise on the level of F4/80 mRNA in lung tissue of mice with lung cancer (n=8). E: Immunofluorescence staining of F4/80 (scale bar, 50μm). F: Statistics of the percentage of F4/80-positive cells in the lung tissues of mice in each group (n=3). Note: && represents significant difference compared with Saline group (P<0.01). *, ** respectively represent a significant difference compared with the CON group (P<0.05), (P<0.01).

Our previous findings showed that both the EX group and the HIIT group had corresponding antitumor effects (Figure 1A, B, C) 15. To appreciate their corresponding antitumor mechanisms, we further examined the degree of infiltration of TAMs in lung cancer tissues. Real-time quantitative PCR (RT-PCR) results showed that compared with the Saline group, the mRNA level of F4/80 in the CON group tended to increase (P=0.08). Compared with the CON group, the mRNA level of F4/80 in the EX group was significantly decreased (P<0.05), and the mRNA level of F4/80 in the HIIT group had a trend of decreasing (P=0.07) (Figure 1D). Thus, both EX and HIIT may reduce the infiltration of macrophages in lung cancer tissue. To further clarify the changes in the number of TAMs in lung cancer tissues, we performed immunofluorescence staining of F4/80, but we found that there was no significant difference in the percentage of F4/80 positive cells among the groups (Figure 1E, F).

### Endurance exercise decreases the proportion of M1 TAMs in lung cancer tissue

**Figure 2:**
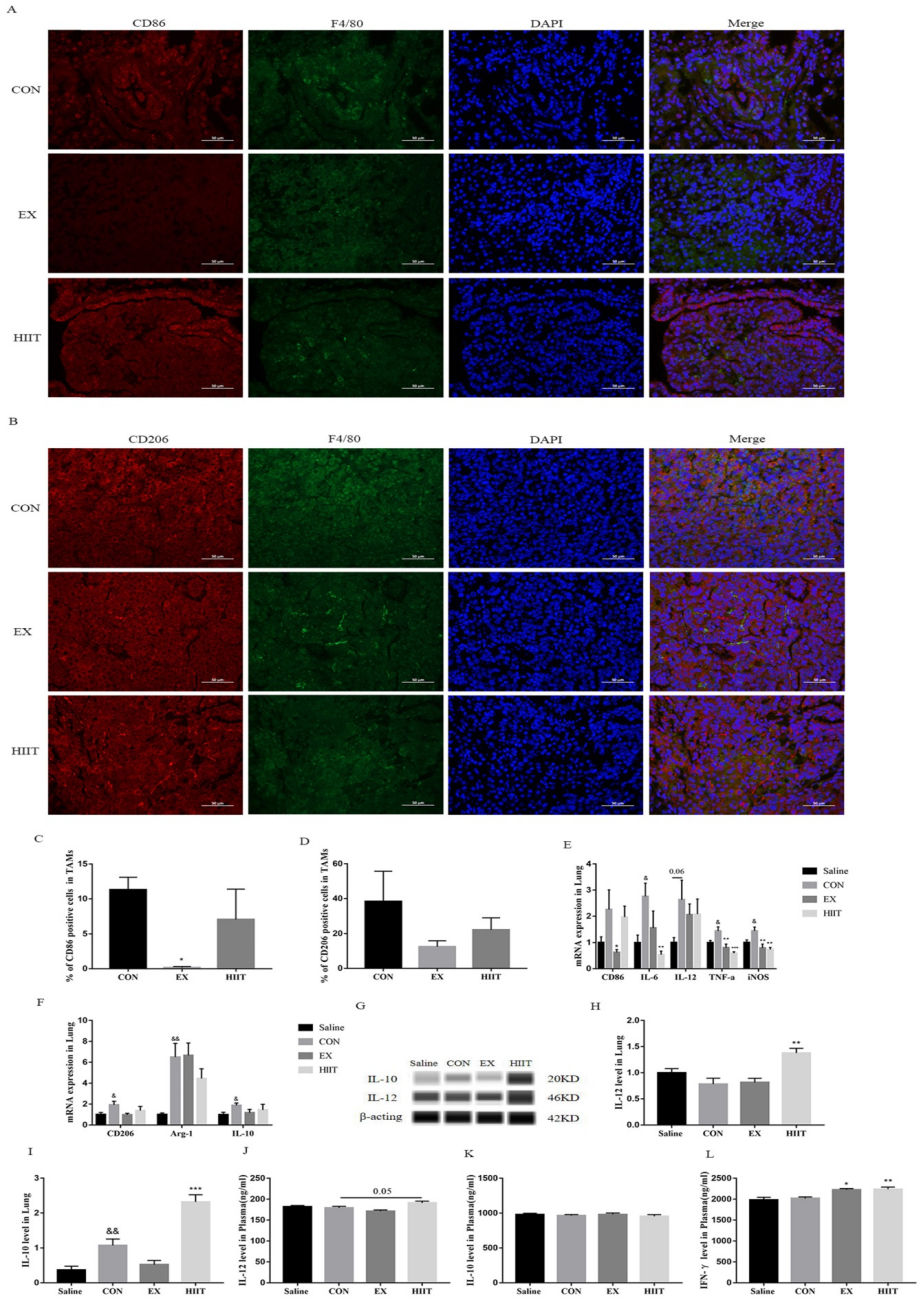
Effects of exercise on the polarization of TAMs in lung cancer tissues. A: CD86 (red) and F4/80 (green) immunofluorescence staining (scale bar, 50μm). B: CD206 (red) and F4/80 (green) immunofluorescence staining (scale bar, 50μm). C: Statistics of the percentage of CD86 positive macrophages in the lung tissues of mice in each group (n=3). D: Statistics of the percentage of CD206 positive macrophages in the lung tissues of mice in each group (n=3). E: mRNA levels of M1 macrophage-related markers (n=8). F: mRNA levels of M2 macrophage-related markers (n=8). G, H: Expression levels of IL-12 in lung cancer tissues (n=6). G, I: Expression levels of IL-10 in lung cancer tissues (n=6). J, K, L: Plasma levels of IL-12(J), IL-10(K) and IFN-γ(L) (n=8). Note: &, && respectively represent a significant difference compared with the Saline group (P<0.05), (P<0.01). *, **, *** represent significant differences compared with CON group (P<0.05), (P<0.01), (P<0.001), respectively.

To explore whether the antitumor effect of exercise is linked with the polarization of TAMs in cancer tissues, we examined the polarization of TAMs in lung cancer tissues. We found that the percentage of CD86+ TAMs in the EX group was significantly lower than that in the CON group (P<0.05) (Figure 2A, C), but there was no significant change in the percentage of CD206+ TAMs (Figure 2B, D). To further confirm this finding, we examined major markers of M1 and M2 macrophages. RT-PCR results showed that compared with the Saline group, the mRNA levels of M1 macrophage markers IL-6, tumor necrosis factor-α (TNF-α), and inducible nitric oxide synthase (iNOS) in the CON group were significantly increased (P<0.05), and IL-12 was elevated (P=0.06)(Figure 2E). Compared with the Saline group, the mRNA levels of M2 macrophage markers CD206 (P < 0.05), arginase-1(Arg-1) (P< 0.01), and IL-10 (P<0.05) were also significantly increased in the CON group (Figure 2F). Notably, the mRNA levels of M1 macrophage markers CD86 (P<0.05), TNF-α (P<0.01), and iNOS(P<0.01) were significantly decreased in the EX group compared with the CON group (Figure 2E). Unfortunately, we did not find that the protein expression level of IL-12 in the EX group decreased in lung cancer tissues. Besides, the mRNA levels of IL-6 (P<0.01), TNF-α (P<0.001), and iNOS (P<0.01) were significantly decreased in the HIIT group compared with the CON group (Figure 2F). In terms of protein expression level, the level of IL-10 in lung cancer tissue of the CON group was significantly higher than that of the Saline group (P<0.01). However, compared with the CON group, the protein expression levels of IL-12 (P<0.01) and IL-10 (P<0.001) in the HIIT group were significantly increased (Figure 2G, H, I). To further clarify the changes of M1-like macrophages and M2-like macrophages in the blood circulation, we tested the levels of plasma IL-12 and IL-10. Compared with the CON group, the level of IL-12 in HIIT was increased (P=0.05), while the level of plasma IL-10 was not significantly different among the groups (Figure 2J, K). Given that IFN-γ favors the M1-like polarization of macrophages 20, we further tested the levels of circulating IFN-γ. The results showed that the levels of plasma IFN-γ were significantly increased in the EX group (P<0.05) and the HIIT group (P< 0.01) compared with the CON group (Figure 2L). In addition, in the spleen tissue, compared with the Saline group, the mRNA level of CD86 in the CON group was significantly decreased (P<0.05), and the mRNA level of ARG-1 was significantly increased (P<0.05), but there was no difference in the mRNA level of CD206. However, compared with the CON group, the mRNA level of IL-12 was increased in the EX group, and the mRNA level of ARG-1 was significantly decreased in the EX and HIIT groups (P<0.01) (Figure S1A). Notably, both the EX group (P=0.08) and the HIIT group (P=0.06) tended to decrease CD206 mRNA levels compared with the CON group (Figure S1B).

### Exercise regulates the expression of immune checkpoints in lung cancer tissues

**Figure 3:**
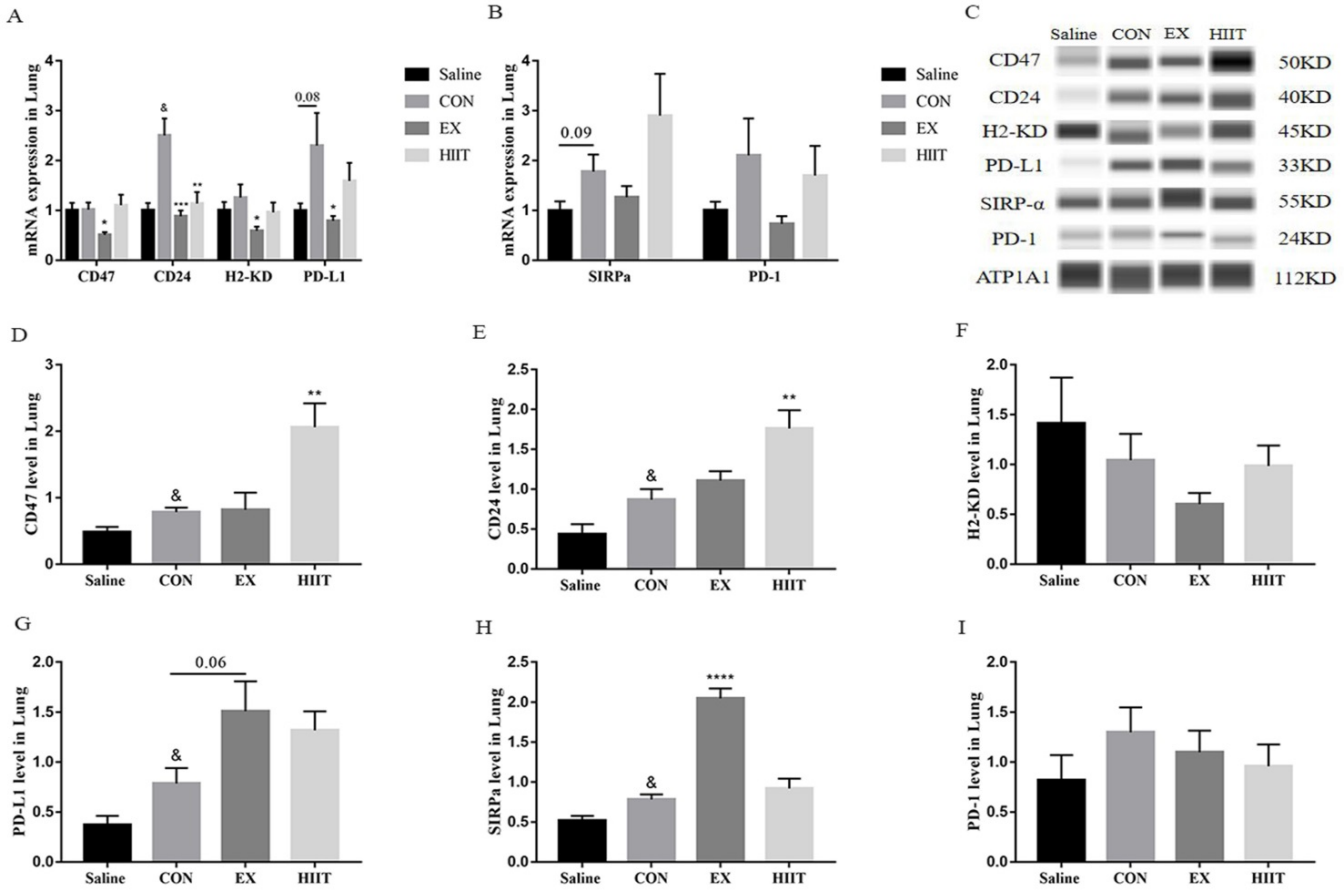
The effect of exercise on the expression of immune checkpoints in lung cancer tissues. A: Tumor cell-associated ligand mRNA expression levels (n=8). B: mRNA expression level of the receptor (n=8). C, D, E, F, G: Expression levels of CD47 (C, D), CD24 (C, E), H2KD (C, F) and PD-L1 (C, G) in lung cancer tissues (n=6). C, H, I: Expression levels of SIRPα (C, H) and PD-1 (C, I) in lung cancer tissues (n=6). Note: & represents significant difference compared with Saline group (P<0.05). *, **, ***, **** represent significant differences compared with CON group (P<0.05), (P<0.01), (P<0.001), (P<0.0001), respectively.

From the above results, we found that the anti-tumor effect of exercise is not by promoting the M1-like polarization of TAMs in lung cancer tissues and inhibiting the M2-like polarization of TAMs. Conversely, endurance exercise reduces the M1-like polarization of TAMs. Therefore, we suspect that exercise may alter the interaction between tumor cells and TAMs. To understand the effect of exercise on the expression of ligands in tumor cells of lung cancer tissue, we detected the mRNA levels of these immune checkpoint genes by RT-PCR. Surprisingly, compared with the Saline group, the mRNA level of CD24 in the CON group was significantly increased (P<0.05), and the mRNA level of PD-L1 tended to increase (P=0.08). Compared with the CON group, the mRNA levels of CD47 (P<0.05), CD24 (P<0.001), H2-KD (P<0.05), and PD-L1 (P<0.05) in the EX group were markedly decreased. The mRNA level of CD24 was also significantly decreased in the HIIT group compared with the CON group (P<0.01) (Figure 3A). Moreover, compared with the Saline group, the mRNA level of SIRPα in the CON group tended to increase (P=0.09) (Figure 3B). However, immunofluorescence experiments showed that compared with the Saline group, the expression level of CD24 in the CON group tended to increase, while compared with the CON group, both the EX and HIIT groups showed a downward trend, but there was no significant difference in these changes (Figure S2A, B). To assess the changes in the expression level of these immune checkpoint proteins in lung cancer tissues, we used the Wes-ProteinSimple system for analysis. In terms of tumor ligand expression, compared with the Saline group, the protein levels of CD47, CD24, and PD-L1 in the CON group were significantly increased (P<0.05). Compared with the CON group, the protein expression level of PD-L1 in the EX group was increased (P=0.06), and the protein levels of CD47 and CD24 in the HIIT group were significantly increased (P<0.01) (Figure 3C, D, E, G). Unfortunately, there was no significant difference in H2-KD expression between groups. From the expression of receptor protein, compared with the Saline group, the expression level of SIRPα in the CON group was significantly increased (P<0.05). Compared with the CON group, the protein expression level of SIRPα in the EX group was considerably increased (P<0.0001) (Figure 3C, H). In addition, the expression of PD-1 was not significantly different among the groups (Figure 3C, I).

## Discussion

TAMs account for ~30-50% of the tumor tissue mass 21. Tumor cells recruit macrophages to infiltrate tumor tissues and then induce differentiation into TAMs, which are then exploited to promote tumor development through multiple strategies 3. It was established that long-term swimming training reduces tumor tissue macrophage infiltration and delays Ehrlich tumor growth in mice 22, suggesting that aerobic exercise may slow tumor growth by inhibiting tumor tissue macrophage infiltration. Similarly, we also found that endurance exercise significantly delayed lung tumor growth in mice. In addition, HIIT also has a certain effect on the treatment of lung cancer (Figure 1B, C). Further study found that compared with the Saline group, the mRNA level of F4/80 in lung tissue of the CON group had a tendency to increase, and endurance exercise could significantly reduce the mRNA level of F4/80 in lung cancer tissue. In addition, HIIT also tended to decrease F4/80 mRNA levels (Figure 1D). However, the results of immunofluorescence staining revealed that there was no significant difference in the rate of F4/80 positive cells in lung cancer tissues among the groups (Figure 1E, F). Notably, it has been observed that 8 weeks of voluntary wheel running does not change the frequency of intra-tumoral macrophages in the subcutaneous I3TC tumor model 23. These findings suggest that whether endurance exercise inhibits macrophage infiltration in tumor tissue depends on the specific cancer type. Therefore, neither endurance exercise nor HIIT could prevent the accumulation of TAMs in lung cancer tissues.

Although a large number of macrophages accumulate in tumor tissues, the macrophages are primarily of the M2 type that facilitates tumor growth, metastasis, and immunosuppression 24. Nowadays, many studies focus on how to target TAMs to reprogram them into M1 macrophages to eliminate tumors. For example, studies have shown that TLR7/8-agonist-loaded nanoparticles can promote the M2 to M1 type switch of macrophages, leading to controlled tumor growth 24. In addition, CpG plus anti-interleukin-10 receptor antibody promptly switch infiltrating TAMs from M2 to M1 type, triggering tumor rejection 25. Therefore, the polarization of TAMs in lung cancer tissues is extremely crucial for antitumor immunity. It was shown that endurance exercise enhances antitumor effects by reducing the mRNA levels of markers of M2 macrophages (CD206, CCL22, and Arg-1) in colon cancer tissue 26. Moreover, endurance exercise promotes the M1-like polarization of macrophages in the peritoneal microenvironment of ovarian cancer mice 27. These findings propose that endurance exercise increases the ratio of M1/M2 macrophages to strengthen their antitumor activity. However, we found that endurance exercise significantly decreased the number of M1-like TAMs in lung cancer tissues (Figure 2A, C), which suggests that the reduced proportion of M1-like macrophages in lung cancer tissues induced by endurance exercise may be detrimental to the recovery of the antitumor activity of macrophages. To appreciate the expression of M1 and M2 TAMs-specific markers in lung cancer tissues, we performed RT-PCR detection on these genes. We found that the mRNA levels of IL-6, TNF-α, and iNOS in the CON group were significantly higher than those in the Saline group, and the mRNA levels of IL-12 in the CON group tended to increase (Figure 2E). The levels of CD206, Arg-1 and IL-10 in the CON group were higher than those in the Saline group (Figure 2F). These results imply that both the pro-inflammatory cytokines of M1-like TAMs and the anti-inflammatory cytokine markers of M2-like TAMs are up-regulated in lung cancer tissues. Indeed, one study found elevated levels of IL-1β and TNF-α in glioblastoma multiforme 28. It was found increased levels of interleukin-10 in serum from patients with hepatocellular carcinoma 29. Indeed, chronic inflammation is now an important hallmark of cancer, and how to improve the inflammatory tumor microenvironment has become an essential direction of tumor therapy today 30. In addition, we found that endurance exercise significantly decreased the mRNA levels of CD86, TNF-α, and iNOS in lung cancer tissues (Figure 2E). This also suggests that endurance exercise improves the inflammatory tumor microenvironment, but it also represses the proportion of M1-like TAMs. Thus, endurance exercise combined with targeting TAMs to reprogram them from M2 to M1 type may be a promising therapeutic strategy for the treatment of lung cancer patients. Notably, endurance exercise did not modify the expression level of IL-12 protein in lung cancer tissue (Figure 2G, H) and its underlying molecular mechanism remains unknown.

So far, there are no other studies on the polarization of TAMs in tumor tissues by HIIT. We found that although HIIT did not down-regulate the mRNA level of CD86, it significantly down-regulated the mRNA levels of IL-6, TNF-α, and iNOS in lung cancer tissues (Figure 2E), which also functions as an anti-inflammatory role. However, at the protein level, we found that HIIT remarkably up-regulated the levels of IL-12 in lung tissue and plasma in the CON group (Figure 2G, H, J). Of note, the increased level of IL-12, which results in IFNγ secretion by effector cells in tumors, is beneficial to initiate innate and adaptive immunity to fight tumors 31. IFN-γ is primarily produced by activated NK cells and NKT cells, as well as CD4+ T cells and cytotoxic CD8+ lymphocytes 32. Indeed, IL-12 can increase IFN-γ secretion in NK cells and T cells 33. Therefore, it is not difficult to speculate that HIIT may activate the innate and adaptive immunity of lung cancer tissue by increasing the level of IL-12 in lung cancer tissue. However, we did not find that HIIT causes the increased number of M1-type TAMs in lung cancer tissue, suggesting that the elevated IL-12 in lung cancer tissue is primarily derived from other immune cells, and the specific mechanism needs to be further clarified. In addition, we found that HIIT increased plasma IFN-γ levels (Figure 2L), which thus may be tied to elevated levels of IL-12 in lung cancer tissues. Furthermore, IFN-γ can induce monocytes to differentiate into immunostimulatory M1 macrophages rather than immunosuppressive M2 macrophages 20. Therefore, HIIT is favorable to the M1 polarization of TAMs and the anti-tumor immunity of other immune cells, which may be an important reason for the therapeutic effect of HIIT on lung cancer. In addition, despite the fact that endurance exercise increased the level of plasma IFN-γ, it decreased the number of M1-type TAMs in lung cancer tissues (Figure 2A, C, L), and the specific mechanism is not clear. Notably, IL-10 also triggers the M2 polarization of macrophages 34. Notably, high levels of IL-10 in lung cancer tissues have been reported 35. Furthermore, both tumor cells and TAMs secrete high levels of IL-10, which promotes tumor cell proliferation and migration 35, 36. We found that lung cancer tissue had significantly higher levels of IL-10 protein than healthy lung tissue, and HIIT also up-regulated the level of IL-10 in lung cancer tissue (Figure 2I). These results suggest that HIIT has a bidirectional regulation effect on TAMs polarization in lung cancer tissue, and the combination of HIIT combined with targeting IL-10 in lung cancer tissue may be among the promising approaches for lung cancer treatment.

We found that from the spleen tissue, compared with the Saline group, the mRNA level of CD86 in the CON group was significantly decreased, and the mRNA level of Arg-1 in the CON group was significantly increased, suggesting that the ratio of M1/M2 macrophages in the spleen of lung cancer mice are decreased, forming an immunosuppressive microenvironment. However, endurance exercise significantly increased the mRNA level of IL-12, significantly decreased the mRNA level of Arg-1, and it had a trend of decreasing the mRNA level of CD206 in the spleen of lung cancer mice (Figure S1A, B), suggesting that endurance exercise promotes the conversion of spleen tissue macrophages from M2 to M1 type. Therefore, the key for endurance exercise to delay tumor growth in mice with lung cancer may lie in macrophages in spleen tissue. In addition, we also found that high-intensity intermittent exercise down-regulated the mRNA level of Arg-1 in the spleen tissue of lung cancer mice, and tended to decrease the level of CD206 mRNA (Figure S1B), but it did not increase the expression of macrophage M1-type markers. Therefore, another mechanism of HIIT in the treatment of lung cancer may lie in reducing the number of M2 macrophages in the spleen, and the specific mechanism remains unclear.

A variety of ligands, highly expressed on the surface of tumor cells, interact with macrophages to inhibit the phagocytosis of macrophages. For example, CD47 highly expressed on tumor cells represses the phagocytic activity of macrophages by interacting with the receptor SIRP-α on the surface of macrophages 37. Moreover, tumor cells also highly express CD24, which binds to SIGLEC10 on the surface of macrophages to inhibit the activity of macrophages 38. Tumor cells utilize the MHC I/LILRB1 axis as well as the PD-L1/PD-1 axis to inhibit the phagocytic activity of macrophages 39, 40. Therefore, we detected the expression levels of ligands and corresponding macrophage receptors in lung cancer tissues. As expected, at the protein expression level, we found that the expression of CD47 and CD24 in lung tissue of lung cancer mice was significantly increased compared with healthy mice. Unfortunately, endurance exercise did not change the expression levels of CD47 and CD24, and HIIT significantly increased the expression of CD47 and CD24 in lung cancer tissues (Figure 3C, D, E), suggesting that HIIT may enhance the inhibitory effect of tumor cells on the phagocytosis of TAMs. In addition, there was no significant difference in the protein level of H2-KD between the groups (Figure 3C, F), suggesting that exercise can not modulate the phagocytic activity of TAMs in lung cancer tissues through MHC I/LILRB1 signaling axis. Notably, the level of SIRPα in lung cancer tissue was significantly higher than that in healthy lung tissue, while endurance exercise also significantly up-regulated the level of SIRPα in lung cancer tissue (Figure 3C, H). The enhanced expression of SIRPα in macrophages is related to the weakening of its phagocytic ability 41. Therefore, endurance exercise-induced elevated SIRPα in lung cancer tissues is associated with a decrease in the tumor phagocytic ability of TAMs. Besides, increased PD-1+ tumor-infiltrating lymphocytes in patients with classical Hodgkin lymphoma are associated with reduced overall survival 42. In addition, the increase of PD-1+ macrophages in gastric cancer tissue is intimately associated with the poor prognosis of gastric cancer patients 43. Unfortunately, the expression of PD-1 was not statistically significant between the groups (Figure 3C, I). Surprisingly, studies have found that voluntary running wheel exercise increases the expression of the immune checkpoints PD-1 and PD-L1 in tumors, which enhances tumor immunogenicity to halt tumor growth 44. Of note, the reduced expression of PD-L1 in NSCLC tumors associates with the immunologic cold tumor microenvironment 45. These findings suggest that the increased PD-L1 in tumor tissue is a double-edged sword, which is beneficial to tumor progression and also contributes to anti-tumor immunity. A clinical study (NCT01295827) found that after all patients with advanced melanoma were treated with pembrolizumab PD-1, patients with high PD-L1 expression had a higher objective response rate, and longer progression-free survival (PFS) and OS 46. Furthermore, PD-L1 expression in tumors is positively correlated with the number of tumor-infiltrating lymphocytes 47, indicating that cancer patients with high intratumoral PD-L1 expression are more likely to respond to immune checkpoint therapy. We found that the expression level of PD-L1 in lung cancer tissue was significantly higher than that in healthy lung tissue, while endurance exercise tended to increase PD-L1 expression in lung cancer tissue (P=0.06) (Figure 3C, G), suggesting that endurance exercise combined with targeting PD-L1/PD-1 axis may be an effective measure to treat lung cancer mice. Unfortunately, it has been found that endurance exercise combined with targeting the PD-L1/PD-1 axis does not further delay melanoma growth 44, which may be related to tumor cell types, and the mechanism remains unclear. Studies have found that IFN-γ can induce the expression of PD-L1 in A549 lung cancer cells through activating the Janus kinase/signal transducer and activator of transcription 3 (JAK/STAT3) signaling and the phosphatidylinositol 3-kinase (PI3K)/AKT signaling 48. Additionally, increased PD-L1 levels are closely associated with increased IFN-γ levels 44. Therefore, endurance exercise may enhance antitumor immunity by increasing the level of IFN-γ to induce PD-L1 expression in lung cancer tissue, thereby enhancing the immunogenicity of lung cancer tissue and turning cold tumors into hot tumors to enhance anti-tumor immunity. Of note, although HIIT increased the level of circulating IFN-γ, it did not increase the expression of PD-L1 in lung cancer tissues (Figure 2L, 3C, G), and the mechanism remains unclear. In addition, HIIT significantly up-regulates the expression levels of CD47 and CD24 in lung cancer tissue (Figure 3C, D, E), similar to PD-L1, which may also be relevant to the inhibition of macrophage phagocytic activity and the enhancement of tumor immunogenicity. Overall, HIIT combined with targeting IL-10, CD47 and CD24 could be a novel strategy for the treatment of lung cancer. Endurance exercise combined with targeting PD-L1, SIRPα and reprogramming TAMs from M2 to M1 type may be an effective strategy for immunotherapy in lung cancer patients.

It has been found that endurance exercise promotes apoptosis in lung cancer tissues of A549 xenograft nude mice by increasing the expression of p53, Bax, and active caspase-3 17. We have also previously found that both endurance exercise and HIIT can inhibit the proliferation of lung cancer cells to delay the progression of lung cancer 15. In this study, we found that endurance exercise can reduce the proportion of M1-type TAMs in lung cancer tissues, while HIIT antagonistically regulates M1 and M2 polarization of TAMs by increasing the levels of IL-10 and IL-12 in lung cancer tissues and circulating IFN-γ. Moreover, endurance exercise and HIIT can modulate the expression of some immune checkpoints in lung cancer tissues. Collectively, these findings may provide new therapeutic strategies for the treatment of lung cancer patients.

## Supplementary Material

Supplementary figures.

## Figures and Tables

**Table 1:** Benefits and mechanisms of exercise for lung cancer

Species	Type	Results	Underlying mechanism	References
Human	Aerobic exercise	Improvement of lung cancer symptoms	Unknown	(Temel et al., 2009) 12
Human	Moderate to vigorous intensity leisure time physical activity	Reducing the mortality of lung cancer patients	Unknown	(Arem et al., 2014) 13
Human	Moderate to vigorous intensity leisure time physical activity	Exercise is associated with better overall survival (OS)	Unknown	(Ha et al., 2021) 14
Mouse	Aerobic exercise	Slowing the progression of lung cancer	Ki67↓ MMP9↓	(Ge et al., 2022) 15
Mouse	HIIT	Slowing the progression of lung cancer	Ki67↓ MMP2↓	(Ge et al., 2022) 15
Mouse	HIIT	Diminishing the incidence of lung tumors	Unknown	(Paceli et al., 2012) 16
Mouse	Aerobic exercise	Slowing the progression of lung cancer	p53↑ ; Bax↑ ; active caspase 3↑ ; Apoptosis in lung cancer tissue↑	(Higgins et al., 2014) 17

## Abbreviations

TAMs: tumor-associated macrophages
SIRPα: signal regulatory protein-α
SIGLEC10: sialic acid binding Ig-like lectin 10
MHC-I: major histocompatibility complex class I molecules
HIIT: high-intensity interval training
LILRB1: leukocyte immunoglobulin-like receptor subfamily B member 1
EX: endurance exercise
PD-1: programmed cell death protein-1
PD-L1: PD-1 ligand 1
OS: overall survival
Arg-1: arginase-1
TNF-α: tumor necrosis factor-α
iNOS: inducible nitric oxide synthase
