# Supplementary material for: Exercise modulates polarization of TAMs and expression of related immune checkpoints in mice with lung cancer
Source: J Cancer. 2022 Sep 6;13(12):3297–307. doi: 10.7150/jca.76136 (PMC9516014; doi:10.7150/jca.76136)
Supplement: Supplementary file 1 — Supplementary figures. [file jcav13p3297s1.pdf]

A

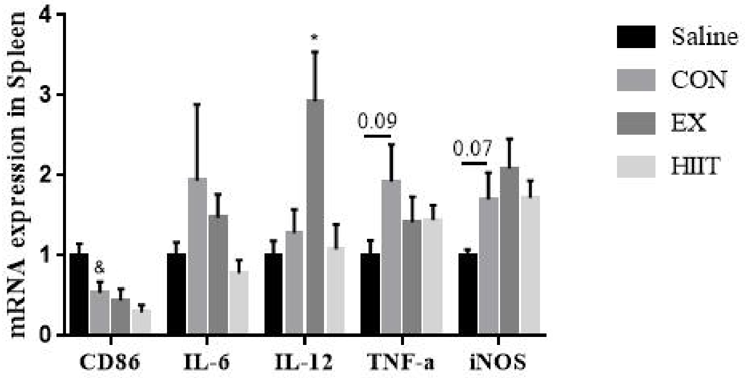

B

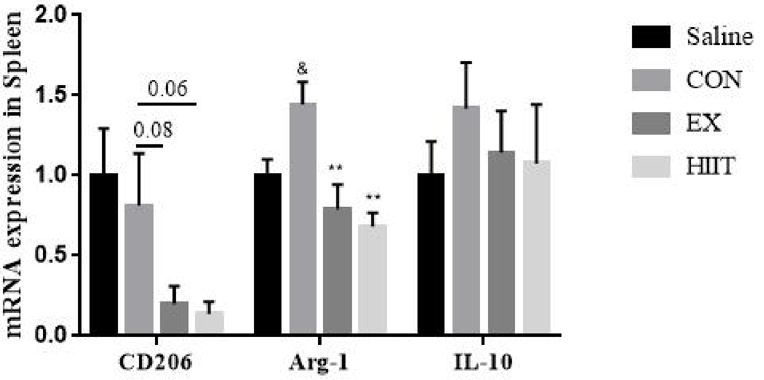

Figure S1：Effects of exercise on the polarization of TAMs in spleen. A: mRNA levels of M1 macrophage-related markers (n=8). B: mRNA levels of M2 macrophage-related markers (n=8). & represents a significant difference compared with the Saline group (P<0.05). *, ** respectively represent a significant difference compared with the CON group (P<0.05), (P<0.01).

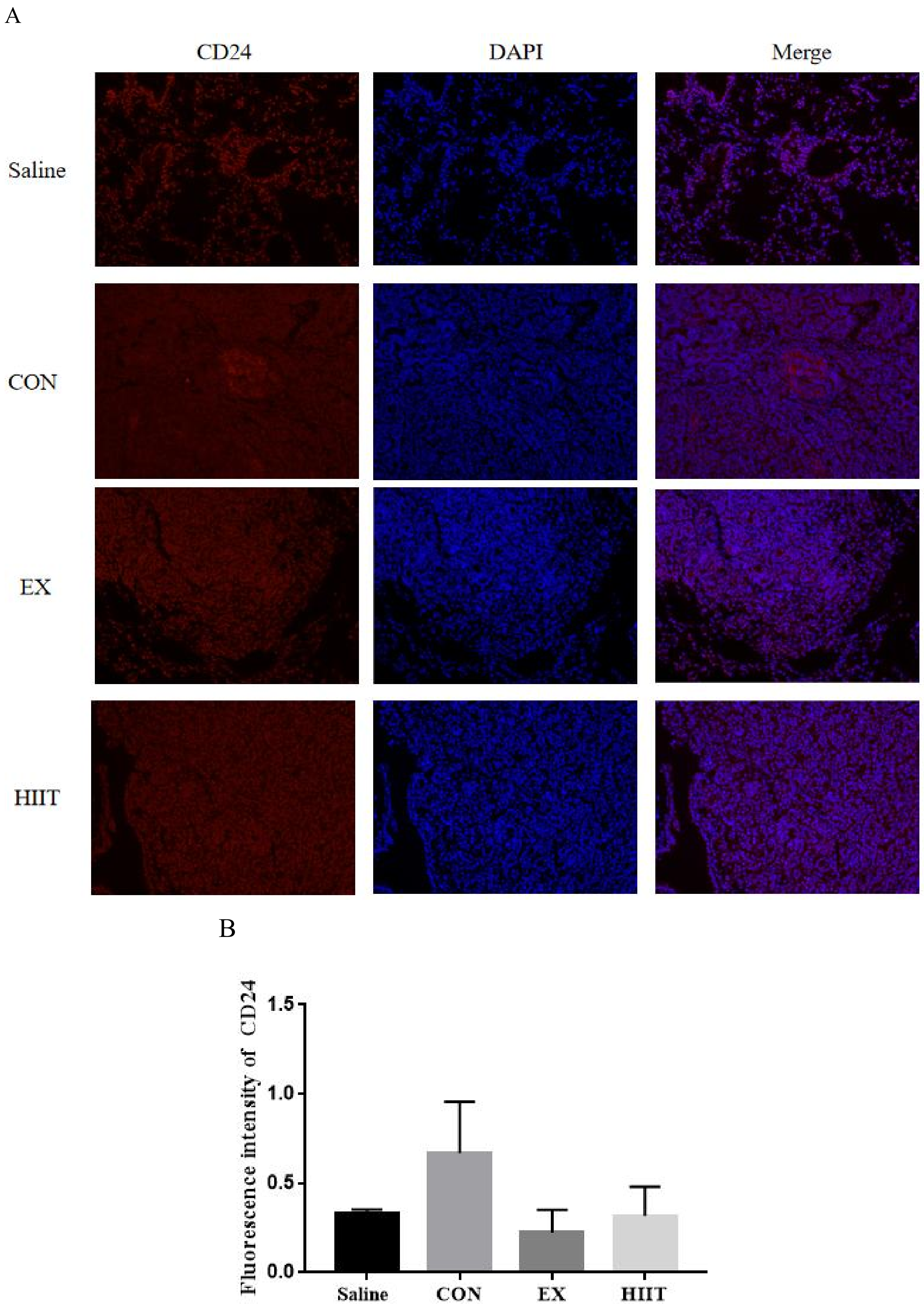

Figure S2: A: Immunofluorescence staining of CD24. B: Fluorescence intensity statistics of CD24 immunofluorescence staining (n=3).
